# Histone deacetylase inhibitors mediate DNA damage repair in ameliorating hemorrhagic cystitis

**DOI:** 10.1038/srep39257

**Published:** 2016-12-20

**Authors:** Subhash Haldar, Christopher Dru, Rajeev Mishra, Manisha Tripathi, Frank Duong, Bryan Angara, Ana Fernandez, Moshe Arditi, Neil A. Bhowmick

**Affiliations:** 1 Department of Medicine, Samuel Ochin Comprehensive Cancer Institute, Cedars-Sinai Medical Center, Los Angeles, CA, USA; 2 Division of Urology, Cedars-Sinai Medical Center, Los Angeles, CA, USA; 3 Greater Los Angeles Veterans Administration, Los Angeles, CA, USA; 4 Department of Pediatrics, Cedars-Sinai Medical Center, Los Angeles, CA, USA

## Abstract

Hemorrhagic cystitis is an inflammatory and ulcerative bladder condition associated with systemic chemotherapeutics, like cyclophosphomide. Earlier, we reported reactive oxygen species resulting from cyclophosphamide metabolite, acrolein, causes global methylation followed by silencing of DNA damage repair genes. Ogg1 (8-oxoguanine DNA glycosylase) is one such silenced base excision repair enzyme that can restore DNA integrity. The accumulation of DNA damage results in subsequent inflammation associated with pyroptotic death of bladder smooth muscle cells. We hypothesized that reversing inflammasome-induced imprinting in the bladder smooth muscle could prevent the inflammatory phenotype. Elevated recruitment of Dnmt1 and Dnmt3b to the *Ogg1* promoter in acrolein treated bladder muscle cells was validated by the pattern of CpG methylation revealed by bisulfite sequencing. Knockout of *Ogg1* in detrusor cells resulted in accumulation of reactive oxygen mediated 8-Oxo-dG and spontaneous pyroptotic signaling. Histone deacetylase (HDAC) inhibitor, suberoylanilide hydroxamic acid (SAHA), restored *Ogg1* expression in cells treated with acrolein and mice treated with cyclophosphamide superior to the standard of care, mesna or nicotinamide-induced DNA demethylation. SAHA restored cyclophosphamide-induced bladder pathology to that of untreated control mice. The observed epigenetic imprinting induced by inflammation suggests a new therapeutic target for the treatment of hemorrhagic cystitis.

Hemorrhagic cystitis is the severe clinical manifestation of several systemic chemotherapeutics, most notably cyclophosphamide (CPX) and other nitrogen mustard alkylating agents^1^,^2^. The primary mechanism of the life-threatening hemorrhage associated with this disease process is sloughing of the urothelium and erosion into the underlying lamina and detrusor vasculature. Acrolein, a corrosive metabolic breakdown product of CPX, is filtered by the kidneys and excreted into the urine where it concentrates in the bladder^3^. The prolonged exposure of the urothelial cells to acrolein leads to a bladder inflammatory process called pyropototic cell death that has been previously described^4^. 2-mercaptoethane sulfonate sodium, commonly referred to as mesna, is administered with CPX to bind and neutralize acrolein in the bladder to limit hemorrhagic cystitis^5^. However, the development of hemorrhagic cystitis 10–20 years after CPX therapy, in for example childhood lymphoma patients, motivated us to consider a mechanism of epigenetic memory in the bladder detrusor^6^.

Inflammation involves aberrant epigenetic alterations through methylation of DNA and histone de-acetylation. Such histone modifications recruit DNA methyltransferases, mediate DNA methylation, and regulate expression of genes implicated in pathology^7^. Promoter cytosine methylation in CpG dinucleotide islands is associated with transcriptional repression^8^,^9^,^10^. Establishment of new DNA methylation is catalyzed by two *de novo* DNA methyltransferase enzymes, DNMT3A and DNMT3B, patterns maintained by DNMT1 as it acts on daughter strands during DNA replication^11^,^12^. We previously reported CPX exposure caused global methylation in mouse bladder detrusor muscle and silenced several DNA damage repair genes associated with pyroptotic cell death^4^. DNA methylation is coupled with histone deacetylation. Histone deacetylases (HDACs) recruitment potentiate local chromatin condensation and gene silencing^13^,^14^. *Ogg1* in particular recognizes 8-oxoguanine (8-oxo-dG), a mutagenic DNA-base byproduct that forms as a result of reactive oxygen species exposure^15^,^16^,^17^. CPX mediated bladder inflammation potentiated mitochondrial DNA oxidation is found to be a substrate for NLRP3 activation and pyroptotic cell death^18^. Pyroptotic cell death of macrophage is associated with a bivalent signaling cascade that results in the generation of IL-1ß and IL-18 enable the recruitment of immune infiltrate^19^,^20^. These signaling cascades are mediated by inflammasomes, molecular platforms that are activated against various types of cellular infections or stress. Signal I of the pyroptotic pathway involve toll-like receptor activation that initiates IL-1ß and IL-18 transcriptional expression by NF-κB stimulation. Subsequently, the signal II cascade can involve NLRP3 binding of oxidized/damaged DNA for the stimulation of the interleukin converting enzyme (caspase-1) in cleaving the precursor peptides of IL-1ß and IL-18 into mature proteins for secretion^21^,^22^. We found that the Ogg1 enzyme can inhibit 8-oxo-dG accumulation and prevent NLRP3 activation in the detrusor. These studies describe the downstream mechanism where detrusor pyroptosis results in bladder hypertrophy and hyperplasia downstream of IL-1ß signaling.

The aim of this study was to examine how the bladder *Ogg1* gene is regulated in cell culture and animal models of hemorrhagic cystitis. We found that bladder smooth muscle cells exposed to acrolein and mouse bladders exposed to CPX cause *Ogg1* promoter DNA methylation for the down regulation of gene expression. The ensuing accumulation of damaged DNA resulted in the activation of NLRP3 for downstream cleaved-caspase 1 and IL-1ß expression in the bladder tissue. We found that the DNA base excision repair gene, *Ogg1*, implicated in pyroptosis, is epigenetically silenced in a model of hemorrhagic cystitis^4^. Providing the standard of care, mesna, during CPX treatment did not prevent the epigenetic changes. However, HDAC inhibition was effective in restoring DNA damage repair, reprogramming the detrusor, and preventing hemorrhagic cystitis.

## Results

**Figure 1:**
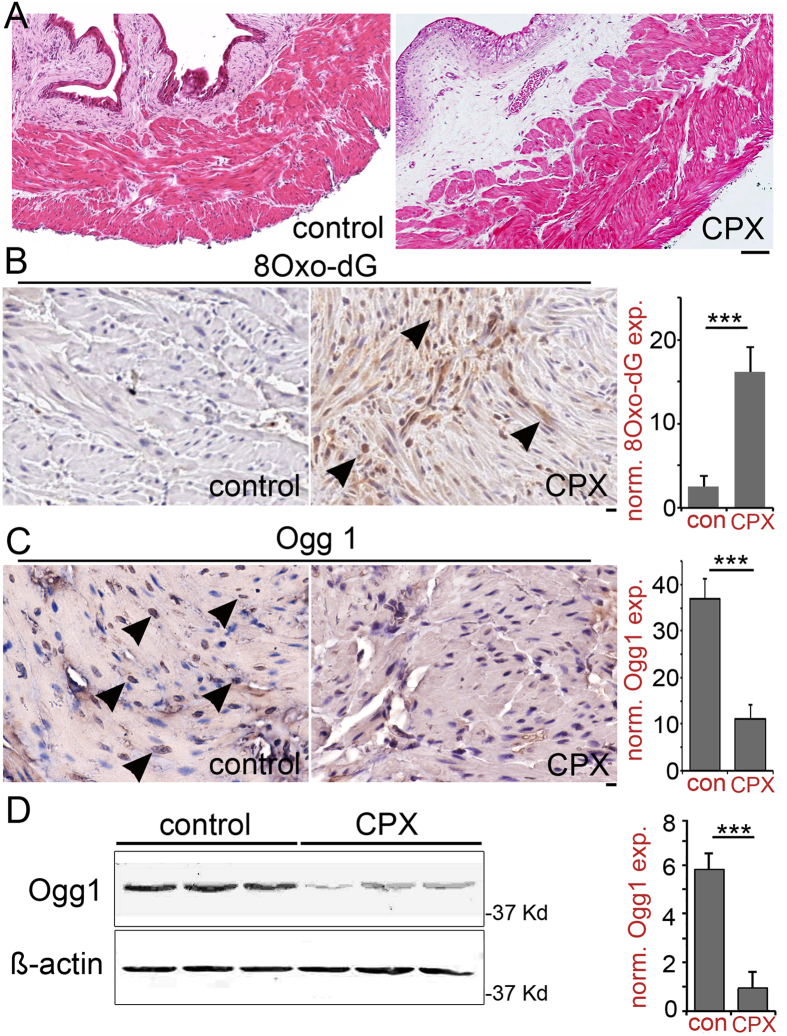
Bladder inflammation associated with Ogg1 down regulation and DNA damage. (**A**), Bladder inflammation was induced in mice by treated with CPX. Bladder inflammation was determined by hematoxylin and eosin (H&E) staining (the *scale bar* represents 64 μm). Immunohistochemical localization of (**B**), 8-Oxo-dG and (**C**), Ogg1 is identified in the detrusor muscle (arrowheads) in control and mouse treated with CPX. The quantitation of the differential staining reached significance (**p value < 0.01; ***p value < 0.001, between groups by student’s T-test, n = 3). The scale bar represents 32 μm. (**D**) Ogg1 protein expression in bladder tissues was measured by Western blotting with ß-actin loading control. The densitometry of the blots indicate relative expression, normalized to ß-actin and mean fold change over control (n = 3).

The established model of hemorrhagic cystitis using CPX treatment is reproducible and amenable to therapeutic intervention in mice^23^,^24^,^25^. H&E bladder sections of CPX-treated mice demonstrated statistically significant urothelial ulceration, expansion of the lamina propria, and detrusor hypertrophy when compared to control (H&E histological score 4.5 ± 0.5 versus 0, p < 0.0001, Fig. 1A). Damaged DNA often manifest in its oxidation in hypertrophic muscle^26^. Immunohistochemical localization of 8-Oxo-dG in bladder tissue of mice treated with CPX suggested reactive oxygen mediated DNA oxidation (Fig. 1B). The DNA repair enzyme, Ogg1, was concomitantly down regulated in the detrusor of CPX treated mice (Fig. 1C). Western blotting of the bladder tissue of the same control and CPX treated mice allowed for quantitation of the Ogg1 expression. Ogg1 expression was down regulated approximately five-fold in the bladders of CPX treated mice, compared to vehicle treated control (Fig. 1D, p value < 0.001). The results suggested a causal relationship of CPX-induced Ogg1 down regulation and accumulation of oxidized DNA.

The inflammatory changes in the bladder from CPX treatment is associated with smooth muscle cell death. The simultaneous treatment with mesna and CPX predictably ameliorated the histologic changes associated with CPX treatment alone. Based on the hypothesis of epigenetic regulation and observed detrusor memory of an inflammatory insult, we treated the mice with nicotinamide, a potent DNA demethylating agent. The combined treatment of nicotinamide and CPX reduced histologic inflammatory changes compared to CPX alone (Supplemental Fig. 1A). Mouse bladder muscle cells treated with acrolein, in a time course of six hours, demonstrated appreciable cell death (Supplemental Fig. 1B). The parallel administration of nicotinamide with acrolein dramatically reduced the visible cell death associated with acrolein exposure in the same time course. Although, there was no significant change in apoptosis by annexin V cell surface expression, FACS analysis for 7AAD staining demonstrated elevated cell death with acrolein treatment that was appreciably reduced by the administration of nicotinamide (Supplemental Fig. 1C).

**Figure 2:**
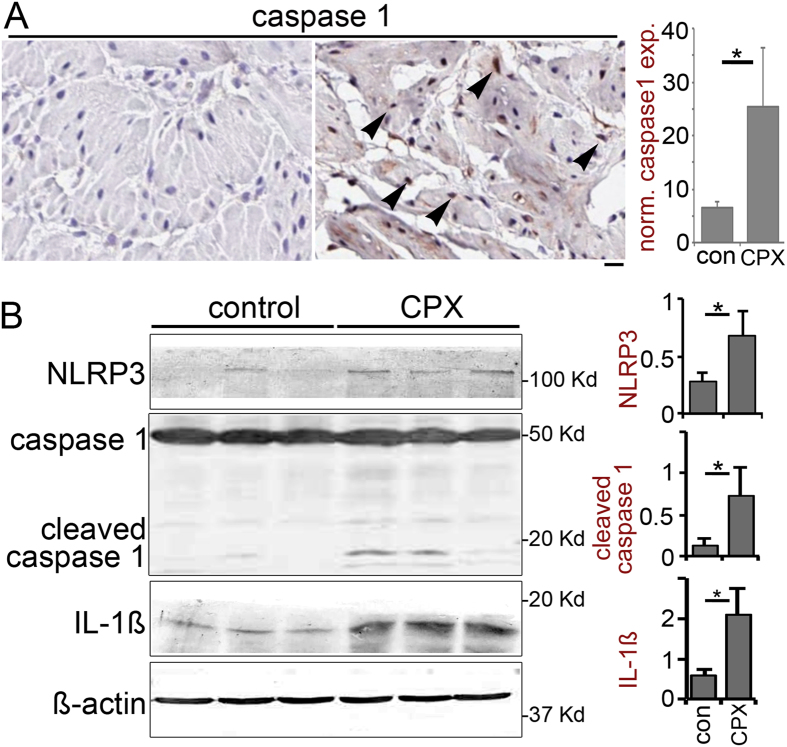
Down regulation of Ogg1 leads to initiation of pyroptotic cascade. (**A**) Caspase 1 was immuno-localized in the detrusor muscle of control and CPX treated mice (arrowheads). There was a significant elevation of caspase 1 staining (scale bare represents 32 μM). The corresponding bar graph illustrate the mean expression of the respective staining (n = 3). (**B**) Immunoblots for NLRP3, cleaved-caspase1, and active-IL-1ß from mouse bladder detrusor muscle tissues in response to CPX treatment were quantitated relative to ß-actin expression (n = 3). Asterisk (*) indicate a p value < 0.05 between groups by Student’s t-test.

Treatment with CPX causes DNA damage and bladder muscle inflammation in a pyroptotic cell death cascade. Western blotting of bladder tissues from saline or CPX treated mice was probed for the expression of inflammasome protein NLRP3. We found that elevated NLRP3 expression and ensuing activation of caspase 1 (cleaved caspase 1) to be associated with the model of hemorrhagic cystitis (Fig. 2A). Immuno-localization of caspase 1 in the detrusor of CPX treated mice supported the Western results of the bladder tissues (Fig. 2B). The tissues from CPX treated mice showed elevated NLRP3 expression and the significant downstream activation of IL-1ß (17 kDa). The induction of mature IL-1ß expression by the bladder muscle is a DNA damage-mediated event potentiated by the NLRP3 activation of caspase 1.

**Figure 3:**
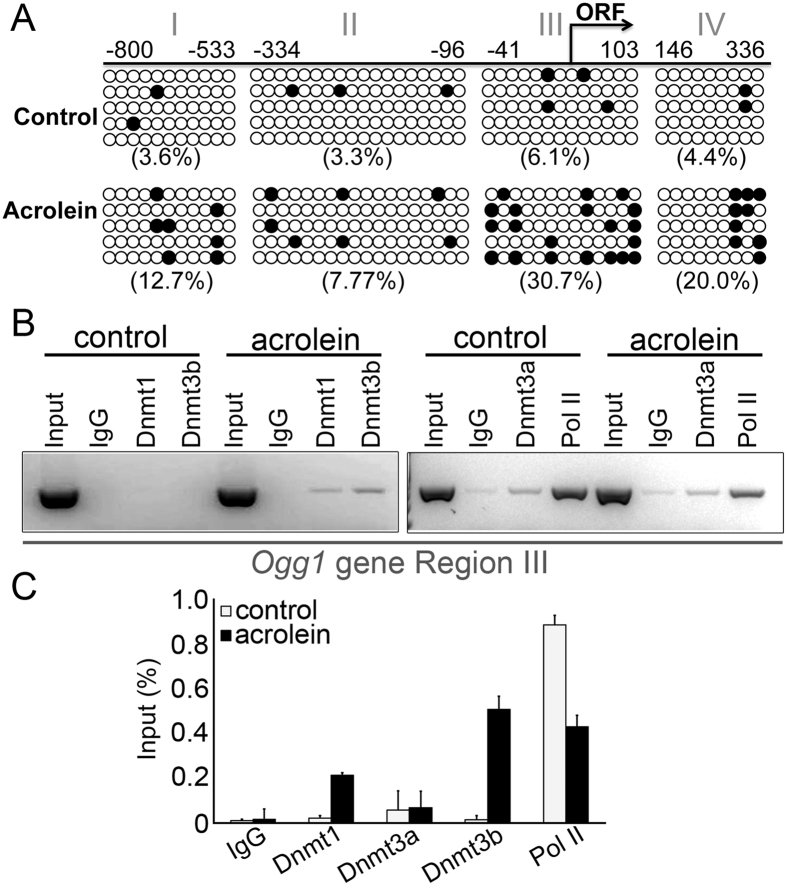
DNA methylation analysis of the Ogg1 gene. (**A**) Bisulfite sequencing was performed on the *Ogg1* gene from promoters region (−800 bp downstream of transcription start site) to exon1 (+336 bp up to transcription start site) from cultured mouse bladder detrusor cells treated with vehicle or acrolein. The CpG islands sequenced fall into regions I, II, III, and IV. The open circles represent the unmethylated cytosine, whereas filled circles represent the methylated cytosine. (**B**) ChIP analysis targeting DNA methylation binding site within the *Ogg1* region III (−41 bp to 103 bp). Dnmt1, Dnmt3A, Dnmt3B, and RNA polymerase II loading onto region III was compared the control and acrolein conditions. Non-immunoprecipitated chromatin was used as total input control. PCR of input DNA shows equivalent starting material for the assay. Normal rabbit IgG antibodies served as the negative control. (**C**) Three independent ChIP assays on region III of the *Ogg1* promoter were analyzed by quantitative PCR for the occupancy of Dnmt1, Dnmt3a, Dnmt3b and Pol II.

Since accumulation of DNA damage in the detrusor is the initiator of the pyroptotic cascade, we wanted to better understand the nature of the epigenetic regulation of *Ogg1* down regulation. Bisulfide sequencing was performed on control and acrolein treated bladder muscle cells with a focus on the specific CpG islands within the *Ogg1* gene promoter and exon1 from −800 bp to +336 bp, including 51 CpG sites (Fig. 3A). Methylation mapping revealed acrolein caused *Ogg1* promoter hypermethylation compared to control cultured bladder muscle cells. An overall 4-fold greater CpG methylation was observed in the acrolein treated cells. The greatest difference in DNA methylation was found near the transcription start site. What we termed as regions III and IV had a five-fold increase acrolein-induced CpG methylation (Fig. 3A). To further determine the mechanism of *Ogg1* DNA methylation, chromatin immune precipitation (ChIP) assays were performed. We focused on Region III (−41 to +103) of the *Ogg1* gene due to its proximity to the transcription start site and site for the greatest hypermethylation. In examining the loading of the proteins responsible for the observed methylation, DNA methyl transferase (Dnmt1 and Dnmt3b) binding to *Ogg1* Region III was greater when treated with acrolein compared to control (Fig. 3B,C). Dnmt3b seemed to have greater binding on Region III compared to Dnmt1 and Dnmt3a. However, there was little difference in the binding of Dnmt3a in the CPX treatment and control groups. The relevance of our focus on Region III was revealed by the differential loading of RNA polymerase II (Pol II). The down regulation of *Ogg1* expression by acrolein treatment was associated with elevated DNA methyltransferase binding and reduced Pol II binding to Region III.

**Figure 4:**
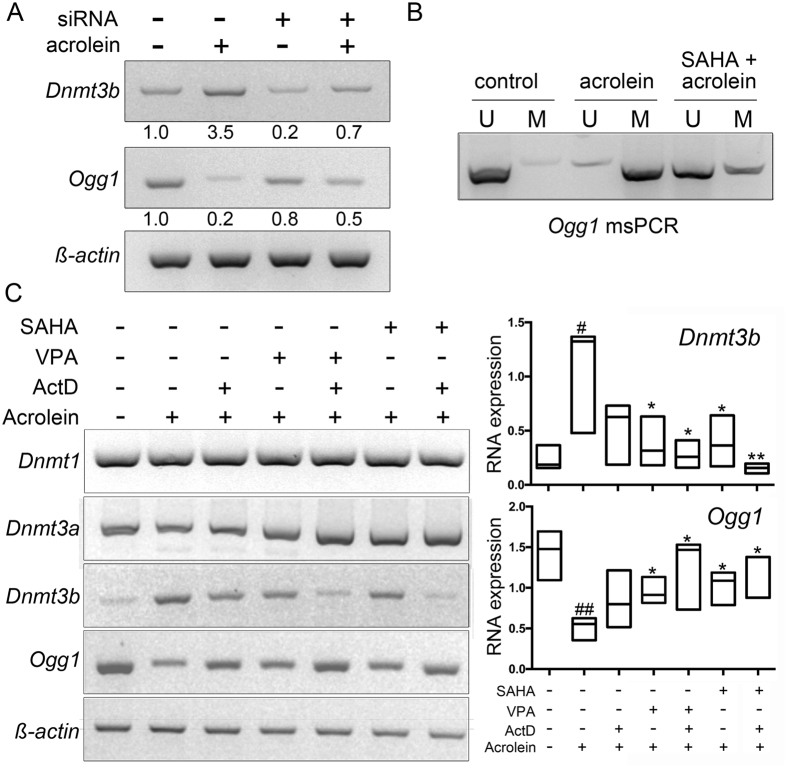
HDAC inhibitors reverse the Ogg1 mRNA expression in mouse bladder muscle cells. (**A**) Bladder muscle cells were transfected with scrambled siRNA or siRNA against mouse *Dnmt3b*. After 48 h, cells were treated with acrolein for 6 h, and harvested to determine Dnmt3b and Ogg1 mRNA expression by rtPCR. (**B**) *Ogg1* promoter methylation status was determined by methylation specific PCR following acrolein treatment in presence and absence of SAHA. Primers specific for the unmethylated (U) and methylated (M) DNA were used. (**C**) mRNA expression of *Dnmt1 Dnmt3a, Dnmt3b,* and *Ogg1* were measured in the presence and absence of VPA, SAHA, actinomycin D, and acrolein in cultured bladder muscle cells. The corresponding graph illustrates the mean and minimum/maximum mRNA expression values of the respective band intensity of *Dnmt3b* and *Ogg1.* The mRNA expressions were quantitated relative to ß-actin expression (n = 3). One way ANOVA analysis between the control and acrolein treatment are indicated by ^##^*p* value < 0.01; ^#^p value < 0.05. Whereas, comparison of the acrolein treatment group and the other groups are indicated by **p value < 0.01; *p value < 0.05.

Chromatin reprogramming by acrolein-insult may regulate Ogg1 expression. The role of Dnmt3b in actrolein-mediated *Ogg1* down regulation was tested. There was a 77% decrease in the levels of *DNMT3b* mRNA expression 72 hours after siRNA transfection (Fig. 4A). In the cells with Dnmt3b knocked down, acrolein-induced *Ogg1* down regulation was limited, compared to control cells treated with acrolein. However, since DNA and histone methylation are coupled processes, we sought to test the role of acrolein on histones in chromatin remodeling. Histone deacetylase (HDAC) inhibitors, alone or synergistically with DNA methyltransferase inhibitors can reactivate epigenetically silenced genes. Methylation specific PCR was performed with bladder muscle cells treated with acrolein in the presence or absence of HDAC inhibitor, SAHA. We found the *Ogg1* gene promoter (Region III) to be highly methylated with acrolein treatment, reversed by SAHA treatment (Fig. 4B). These data suggested that SAHA modify histone acetylation, contribute to the remodeling of DNA methylation patterns, to regulate *Ogg1* expression.

We used two different HDAC inhibitors valporic acid (VPA) and SAHA to further determine if either treatment could reverse silenced *Ogg1* expression. Primary cultures of bladder muscle cells were pretreated with VPA or SAHA for 24 hours before exposure to actinomycin D (to halt RNA synthesis) and acrolein for 6 hours. *Dnmt3b* mRNA expression elevated by acrolein treatment was significantly down regulated by either SAHA or VPA (Fig. 4C). The presence of actinomycin D highlighted the role of SAHA on *Dnmt3b* mRNA stability^27^. As expected, *Ogg1* mRNA expression was inversely proportional to the *Dnmt3b* expression. However, there was no significant mRNA expression change of other DNA methylating enzymes *Dnmt1* and *Dnmt3a*.

**Figure 5:**
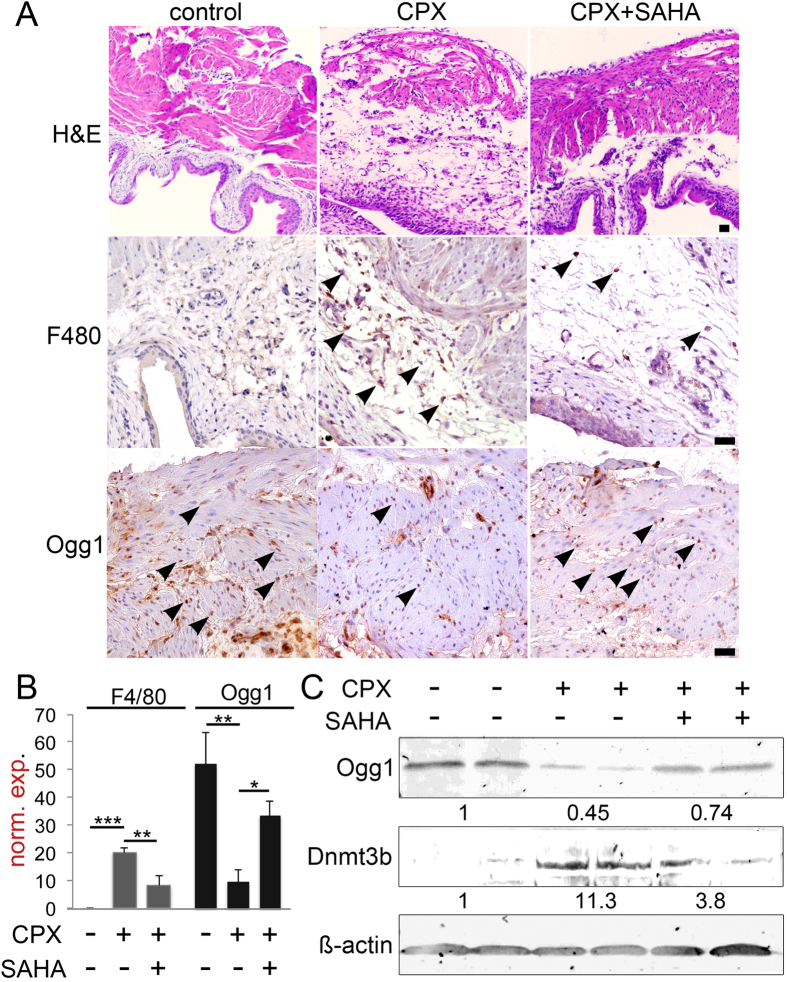
HDAC inhibitors restore Ogg1 expression in mouse bladder. (**A**) Bladder inflammation was determined by H&E staining (the *scale bar* represents 64 μm) and supported by immunohistochemical detection of macrophage by F4/80 staining in the presence or absence of cyclophosphamide and SAHA (arrowheads, the *scale bar* represents 32 μm). Ogg1 expression was localized by IHC (arrowheads). (**B**) Corresponding bar diagram is the quantitation of the differential positive staining of F4/80 and Ogg1 (**p value < 0.01; ***p value < 0.001, between groups by one way ANOVA, n = 3). (**C**) Western blot of two representative bladder tissues from each of the three treatment groups indicate Ogg1 and Dnmt3b expression. The mean quantitations are indicated below each corresponding blot normalized to ß-actin expression.

To ultimately determine the impact of chromatin reprograming we treated the CPX mouse models with SAHA. Strikingly, the bladders of the CPX + SAHA treated mice had histology near that of control mice (Fig. 5A). SAHA significantly down regulated the recruitment of F4/80-expressing macrophage to the bladders compared to that of mice treated with CPX alone (Fig. 5B). In parallel, the down regulation of Ogg1 in the detrusor by CPX was restored to near control levels by co-treatment with SAHA. Western blot analysis of the bladder tissues showed elevation of Dnmt3b expression accompanied with down regulation of Ogg1 in CPX treated group (Fig. 5C). Treatment of the mice with SAHA significantly up regulated Ogg1 expression in the bladder tissues. We observed that SAHA treatment prevented urothelial ulceration and restored detrusor Ogg1 expression epigenetically silenced by CPX.

**Figure 6:**
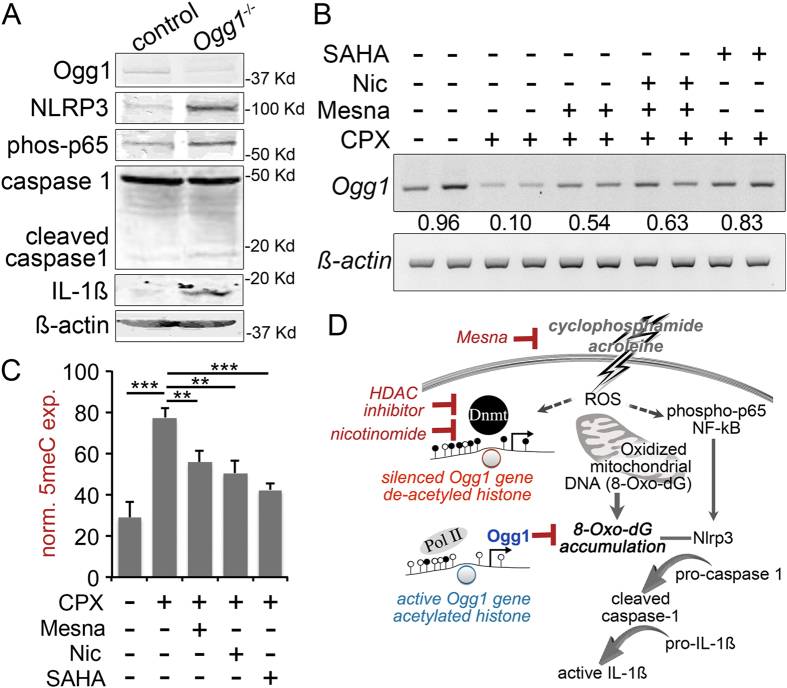
Reactivation of Ogg1 in bladder muscle reduce pyroptosis mediate cell death. (**A**) Immunoblot of cultured bladder muscle cells from wild type and *Ogg1*-knockout mice indicate the expression of affected proteins involved in pyroptotic cell death relative to ß-actin. (**B**) Mice were treated with CPX in the presence or absence of Mesna, nicotinamide (Nic), or SAHA. Representative bladder tissues from mice under the indicated treatments were subjected to rtPCR for *Ogg1* mRNA expression. Mean quantitation of *Ogg1* expression changes are represented below the blot relative to ß-actin expression. (**C**) Immunohistochemical localization of 5meC expression in the detrusor muscle was quantitated as a percentage of total cells per field. Data represent the mean ± S.D. **p value < 0.01; ***p value < 0.001, between groups by one way ANOVA (n = 3). (**D**) ROS induction by cyclophosphamide or acrolein in the bladder muscle can potentiate both NF-κB activation and 8-Oxo-dG accumulation. Mesna serves to sequester acrolein. The *Ogg1* promoter DNA methylation (filled lollipops) by DNMTs is associated with de-acetylated histones and Ogg1 silencing to further 8-Oxo-dG accumulation. HDAC inhibitors and nicotinamide can reverse Ogg1 silencing by DNA de-methylation (open lollipops) and/or histone acetylation to inhibit 8-OxodG accumulation and pyroptotic cell death by way of NLRP3 and caspase1 activation for the expression of mature IL-1ß.

To ultimately determine the impact of *Ogg1* on the pyroptotic cascade, we examined bladder muscle cells from transgenic *Ogg1*-knockout mice. The *Ogg1*-knockout cells are reported to accumulate reactive oxygen^28^. Detrusor cells prepared from *Ogg1*-knockout mice were confirmed to have a loss of Ogg1 expression by Western blotting. Interestingly, NF-κB p65 was also activated in the *Ogg1*-knockout cells, as part of signal I (Fig. 6A). The *Ogg1*-knockout detrusor had elevated NLRP3 expression and downstream cleaved-caspase 1, as components of signal II. The *Ogg1-*knockout muscle cells expressed mature IL-1ß.

Accordingly, we hypothesized that epigenetic reprogramming induced by CPX could be reversed by chromatin modification. We performed a final mouse study comparing the treatment of mesna, nicotinamide, and SAHA in the CPX model. As expected, CPX down regulated *Ogg1* mRNA expression that was partially re-expressed upon co-treatment with mesna (Fig. 6B). However, restoration of *Ogg1* expression was significantly greater with SAHA treatment, compared to either mesna or nicotinamide. The DNA methylation state of the bladder tissues were assessed by IHC for 5-methyl cytosine (5meC). Mesna and nicotinamide significantly limited the DNA hypermethylation induced by CPX in the tissues, but only the levels of 5-meC detected in the SAHA treated group had no statistical difference from control mice (Fig. 6C and Supplemental Fig. 2). Figure 6D summarizes the role of epigenetic regulation of Ogg1 on bladder detrusor pyroptosis identified in hemorrhagic cystitis and potential therapeutic measures.

## Discussion

The prevention of hemorrhagic cystitis induced as a chemotherapeutic side effect could significantly improve the quality of life of cancer patients. The reported latency in the pathology from the initial insult suggested epigenetic memory could be involved^29^. In our previous study, we determined acrolein mediated promoter methylation of *Ogg1*, among six other DNA damage repair genes: *Parp1, Neil1, Rad50, Rad54, BRCA1,* and *Neil2*^4^. We had identified that epigenetic silencing of *Ogg1* contributed to accumulation of DNA oxidation (8-Oxo-dG) in the activation of pyroptosis signal II. The mechanism of *Ogg1* epigenetic silencing by CPX was the focus of this study. *Ogg1* deficiency exhibited tissue-specific increases in both signal I and signal II pyroptotic cascades, associated with the mice in recruiting inflammatory infiltrates and developing hemorrhagic cystitis downstream of IL-1ß expression (Fig. 6D). Reduced Ogg1 expression in the detrusor impaired the repair of acrolein-induced oxidative DNA damage. Of note, we did not observe pyroptotic markers to be expressed in the urothelium. In our previous study, we found that IL-1ß generated through pyroptotic bladder muscle cell death to be responsible for CPX-mediated inflammatory cell recruitment and detrusor expansion^4^. The mechanism of *Ogg1* silencing was demonstrated for the first time through bisulfide sequencing and the epigenetic mediators Dnmt1 and Dnmt3b. Nicotinamide treatment accordingly demonstrated beneficial effects; but, not significantly better than simply sequestering acrolein by mesna administration. It appears that addressing the histone modifications that recruit the DNMTs has a greater effect on global DNA methylation, *Ogg1* expression and ensuing inflammatory cascade. Unfortunately, the CPX mouse model used here is not amenable to longer term studies, to ultimately test if reprogramming the detrusor can be curative. However, the inversely proportional expression of *Ogg1* and the pathologic manifestations of hemorrhagic cystitis described here are supportive of clinical testing.

The epigenetic mediators maintain the balance of accessibility to the promoter by transcription factors through the methylation of CpG islands. We found a 4-fold in CpG methylation in acrolein treated bladder cells, mostly near the transcription start site, where epigenetic changes have a greatest impact. In the examination of DNA methyl transferase recruitment to the *Ogg1* promoter in acrolein treated bladder cells, we found Dnmt1 and Dnmt3b to be preferentially recruited (Fig. 6D). Among the three major DNA methyltransferases, Dnmt1 is the maintenance enzyme, however Dnmt3a and Dnmt3b target unmethylated CpGs to initiate *de novo* methylation^30^,^31^,^32^. That means acrolein treatment both initiates and maintains DNA methylation of *Ogg1*. The prevention of RNA polymerase II recruitment on the *Ogg1* promoter in acrolein treated cells is direct evidence for the observed down regulation of *Ogg1* expression and ensuing accumulation of DNA damage in the detrusor. These findings support *Ogg1* down regulation to be resultant of significant DNA methylation caused by the inflammatory insult caused by CPX.

HDAC inhibitors are often used to investigate epigenetic mechanisms involved in chromatin remodeling and gene expression. We found SAHA and VPA inhibit Dnmt3b expression, reducing *de novo* methylation of the *Ogg1* promoter. In several studies, HDAC inhibitors were capable of inducing DNA de-methylation even in the absence of 5-aza-2′-deoxycytytidine^33^,^34^,^35^,^36^. Here, we showed the mechanism by which HDAC inhibitors reactivate *Ogg1* expression by affecting DNA methylation through their regulation on Dnmt3B. Xiong *et al*. reported that that trichostatin A (TSA), a HDAC inhibitor down-regulates DNMT3B mRNA and protein expression in endometrial cancer cells, resulting in significant decrease in *de novo* methylation activities^27^. They also found that TSA down regulates DNMT3B mRNA stability and reduces its half-life from 4 to 2.5 hours^27^. We found a significant reduction of *Dnmt3b* mRNA and protein level in cultured cells and mouse tissue after SAHA treatment. DNA de-methylating agents may not address the histone modifications that recruit DNMTs chronically. Thus, HDAC inhibition enables DNA de-methylation as well as addresses its canonical histone target to allow Ogg1 expression.

Hemorrhagic cystitis is a morbid condition with associated mortality if not adequately treated in a timely fashion. The clear association of hemorrhagic cystitis with CPX and other nitrogen-mustard alkylating agents has mandated that mesna be administered at the time of treatment. However, mesna is only administered to sequester acrolein immediately before and during cyclophosphamide therapy. However, there are a percentage of patients who develop hemorrhagic cystitis in spite of mesna administration, years after treatment^29^. Like with cyclophosphamide, the etiology of this delayed pathology for radiation-induced cystitis is unknown. We previously demonstrated that a similar pyroptotic cascade in the bladder smooth muscle is induced by radiation^4^. We have outlined a novel epigenetic mechanism for initiation and maintenance of *Ogg1* silencing. The significance of this finding is exemplified in the *de novo* pyroptosis observed in the bladder smooth muscle of *Ogg1*-knockout mice. However, we are unable to rule out Ogg1-independent factors mediated by HDAC inhibition mediating the resolution of bladder inflammation. The role of Ogg1 in pyroptotic cell death in bladder inflammation was previously reported^4^. Other mediators of bladder hypertrophy and hyperplasia such as growth factors and cytokines may be directly epigenetically regulated by HDAC inhibition. Interestingly, those down stream of pyroptotic signaling (e.g. IL-6, IGF1, IL-1ß) are dependent on Ogg1 expression and documented to be consequential to the bladder pathology^37^,^38^,^39^. Future studies could test if long-term prophylactic treatment with HDAC inhibitors following CPX/mesna treatment schedule could reduce the rates of hemorrhagic cystitis in this highly susceptible population. Apart from identifying a potential therapeutic for bladder inflammation, more broadly, the data suggest that reprograming epigenetic imprinting could limit the inflammatory process induced by a toxic insult like CPX.

## Methods

### Animal Experiments

Female C57B/6 mice, aged 9 to 13 weeks, were housed in a pathogen-free environment at the Cedars-Sinai Medical. All mice were ovarectomized. Three weeks later, mice were treated daily with intraperitoneal injections of saline, nicotinamide (30 mg/kg), sodium 2- sulfanylethanesulfonate (mesna, 150 mg/kg) or suberoylanilide hydroxamic acid (SAHA, 50 mg/kg, Torics, Minneapolis, MN). Three days following the initiation of such treatments, hemorrhagic cystitis was induced by intraperitoneal injection of cyclophosphamide 150 mg/kg, as described previously^4^. Paraffin sections of harvested bladders were stained with hematoxylin and eosin. The severity of inflammation was graded on H&E sections in a blinded fashion based on a previously described scoring systems of inflammatory cell infiltration, edema, hemorrhage, urothelial erosion and ulceration (n ≤ 5 per group)^24^.

### Statements of Ethical Approval

The protocol for the animal study was approved by the Institutional Animal Care and Use Committee at Cedars-Sinai Medical Center (# 3679). All experimental protocols were conducted in accordance with institutional regulations and guidelines.

### Cultured Cells

Wild-type and *Ogg1*^−/−^ primary mouse bladder detrusor were cultured in DMEF12 media (Hyclone, Logan, UT) supplemented with fetal bovine serum 5% (Hyclone), Nu serum 5% (Hyclone), 5 μg/ml insulin (Gibco), and gentamycin (Gibco) at 37 °C with 5% CO_2_. Wild-type fibroblasts were grown in the presence of nicotinamide 200 μM (Acros, Canoga Park, CA) for 72 hours before all acrolein treatments. Wild-type cells were incubated with acrolein (25 μM, Sigma-Aldrich, St. Louis, MO) for 0–48 hours and then isolated, as previously described^4^,^24^,^40^. *Ogg1*^−/−^ bladder detrusor cells were incubated with 2% serum containing medium for 48 hours before Western blot was done.

### Immunohistochemisry

Immunohistochemical localization was performed with paraffin-embedded tissue sections, deparaffinized and hydrated through xylene and graded alcohols using a standard protocol. Antibodies are used against Caspase 1 (Santa Cruz Biotechnology, Santa Cruz, CA), 8-oxo-dG (Abcam, Cambridge, MA), and Ogg1 (LSBio, Seattle, WA). Appropriate HRP-conjugated secondary antibodies and DAB incubation (Dako North America, Carpinteria, CA, USA) was used for visualization. All the slides were scanned on a Leica SCN400 (Leica Micro System, Buffalo Grove, IL) and analyzed by Tissue IA Optimizer (Leica). The values of positively stained cells were measured in an unbiased manner.

### Western blotting

Western blots performed with 10, 12, or 15% SDS-polyacrylamide gels were incubated with primary antibodies for IL-1ß (R&D Systems, Minneapolis, MN), NLRP3 (LSBio, Seattle, WA), Ogg1 (Novus Biologicals, Littleton, CO), Caspase 1 (Genetex, Irvine, CA), and DNMT3b (Abcam, Cambridge, MA). Western blots were visualized using alkaline phosphatase-conjugated secondary antibodies (Sigma-Aldrich).

### DNA methylation analysis

Bisulfite treatment was performed on DNA isolated from wild type and acrolein treated cultured mouse bladder fibroblasts using the EZ DNA methylation-Gold kit (Zymo Research, Irvine, CA) according to the vendor’s recommendations. Bisulfite converted DNA was amplified with the primers listed in Supplemental Table 1. We used five primers, which started from 800 bp upstream of the *Ogg1* open reading frame and ended 336 bp downstream of the transcriptional start site. PCR products were cloned in pCR2.1-TOPO vector (Invitrogen, Grand Island, NY) and at least 5 clones from each sample subjected to sequencing through Sanger’s method using M13 primer. Bisulfite converted DNA was used for methylation specific PCR with primers specific for the derivatized-methylated and unmethylated *Ogg1* gene promoter (Supplemental Table1).

### Chromatin immunoprecipitation (ChIP)

ChIP was performed according to the manufacturer’s ChIP protocol (Zymo-Spin CHIP Kit). For each ChIP reaction, immunoprecipitation was performed using 100 μl of sheared chromatin mixed with 10 μg of antibody for Dnmt1, Dnmt3a, Dnmt3b, RNA polymerase II (Abcam, Cambridge, MA) or control IgG along. The purified immunoprecipitated DNA were PCR amplified (see Supplemental Table 1 for primer sequences), and was compared to input DNA, diluted 100-fold. PCR products were resolved by 2% agarose gel electrophoresis.

### Measurement of Dnmt3B mRNA stability

We examine the role valproic acid (VPA, Sigma-Adrich) and SAHA on DNA methyl transferase stability. Mouse bladder fibroblasts were treated for 24 hours with VPA (5mM) and SAHA (10 μM) before actinomycin D (5 μg/mL, Fisher Scientific, Waltham, MA) and acrolein treatment for 6 hours. *Dnmt1, Dnmt3a, Dnmt3b* and *Ogg1* mRNA expression were measured in present and absence of VPA, SAHA, acrolein and actinomycin D by rtPCR (see Supplemental Table 1 for primer sequences). The knockdown of *Dnmt3b* by a pool of siRNA (Santa Cruz Biotechnology) was transfected into primary bladder muscle cells by neucleofection (Lonza, Walkersville, MD).

### Statistical Analysis

Experiments were done a minimum of three times. Results were expressed in terms of mean ± S.D. Student’s t-test and one-way ANOVA were used for comparisons between groups. Statistical analysis was performed using Origin software (OriginLab, Northampton, MA).

## Additional Information

**How to cite this article**: Haldar, S. *et al*. Histone deacetylase inhibitors mediate DNA damage repair in ameliorating hemorrhagic cystitis. *Sci. Rep.* **6**, 39257; doi: 10.1038/srep39257 (2016).

**Publisher's note:** Springer Nature remains neutral with regard to jurisdictional claims in published maps and institutional affiliations.

## Supplementary Material

Supplementary Dataset 1
