# Supplementary material for: Histone deacetylase inhibitors mediate DNA damage repair in ameliorating hemorrhagic cystitis
Source: Sci Rep. 2016 Dec 20;6:39257. doi: 10.1038/srep39257 (PMC5171776; doi:10.1038/srep39257)
Supplement: Supplementary Dataset 1 [file srep39257-s1.pdf]

Histone deacetylase inhibitors mediate DNA damage repair in ameliorating hemorrhagic cystitis

Subhash Haldar, Christopher Dru, Rajeev Mishra, Manisha Tripathi, Frank Duong, Bryan Angara, Ana Fernandez, Moshe Arditi, Neil A. Bhowmick

Supplemental Table 1. List of primers used.

Name	5' -3'	
Bisulfite sequencing Primer 1	Forward	AGTTTTTTTTGGAATAAGGTGGTT
	Reverse	TCAAAAACCAATCTCAAATTTACTC
Bisulfite sequencing Primer 2	Forward	AATTTGAGATTGGTTTTTGAAGTTT
	Reverse	TTCTAAAACAATAATTTTTCAAC
Bisulfite sequencing Primer 3	Forward	TTGTAGGTAGGTTTTGAGATTGTAT
	Reverse	ATTTAACCCTAAAAATAACCAATCC
Bisulfite sequencing Primer 4	Forward	TTGGTTATTTTTAGGGTTAAATAAG
	Reverse	ATCCCTATTAAATAAATTTCCCTCTC
Bisulfite sequencing Primer 5	Forward	AATAGGGATTTAAATTAAAATTATAT
	Reverse	CTCATACTACTAAACAACCAAAAAC
CHIP (RT-PCR) primer for *Ogg1* Promoter	Forward	GGG ACC CGG AAG AAC CAT AC
	Reverse	CCTAGCTTATGGGTCCGGTG
CHIP (Real Time PCR) primer for *Ogg1* Promoter	Forward	CGGTGGGAGTAAACTGGGAC
	Reverse	CCTCGTAGGAAAGCCTCTCG
Methylation specific primer for *Ogg1* Promoter (Un-methylated)	Forward	TTTTTTTAAATTTTGAGGGTTGTGT
	Reverse	AACACCATAAATCCTAACTTATAAATCCA
Methylation specific primer for *Ogg1* Promoter (Methylated)	Forward	TTTTTTTAAATTTTGAGGGTTGTGT
	Reverse	CCGTAAATCCTAACTTATAAATCCG
Ogg1 RT-PCR primer	Forward	GCGAGAGGCTTTCCTACGAG
	Reverse	AGTCCTAAAGCCTCGCACAC
Dnmt1 RT-PCR primer	Forward	CCACCACCAAGCTGGTTA T
	Reverse	TCATCGATGCTCACCTTCTG
Dnmt3a RT-PCR primer	Forward	GGGGACAAGAATGCTACCAA
	Reverse	CTTGTTGTAGGTGGCCTGGT
Dnmt3b RT-PCR primer	Forward	AAGAGCCTCCCCCAGAATCA
	Reverse	TGATGGGGTACTGACGCTCT

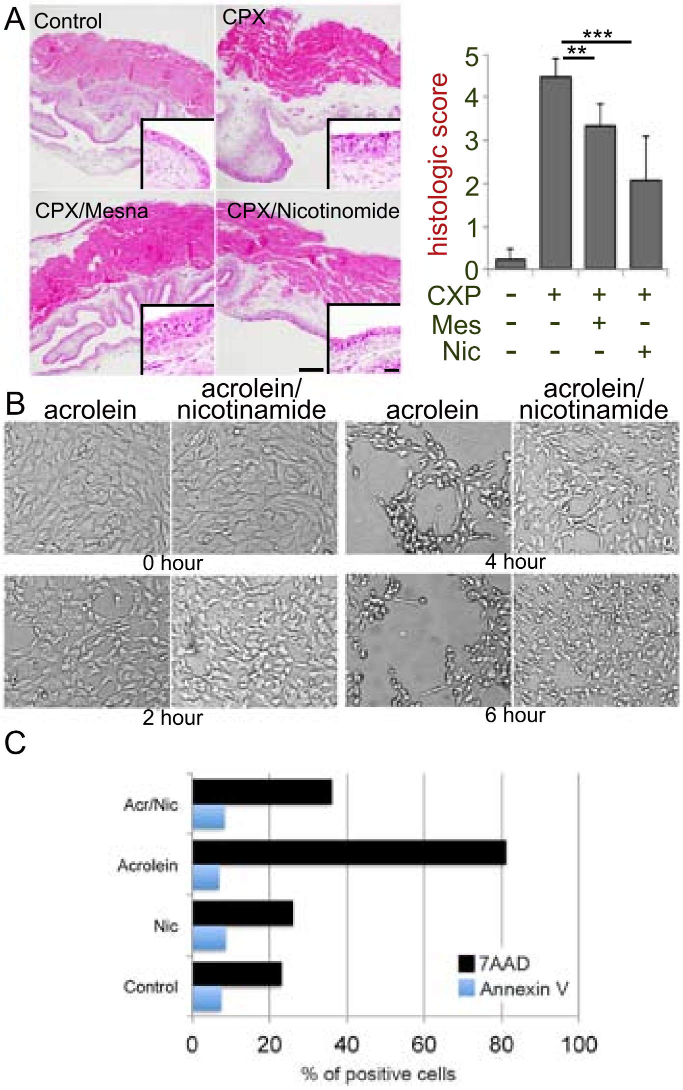

Supplemental Figure 1. ***Histological analysis and acrolein mediated cell death.*** **A)** Histology of bladders from control mice, and those treated with cyclophosphamide (CPX), CPX and mesna (Mes), and CPX and nicotinamide (Nic). Objective histologic grade definition were graphed: 0 - Normal tissue, 1 - Minimal inflammatory infiltration in the lamina propria, 2 - Mild inflammatory infiltration in the lamina propria, 3 - Moderate inflammatory infiltration in the lamina propria with some inflammatory extension into the detrusor, 4 - Moderate inflammation in both the lamina propria and detrusor, 5 - Severe inflammation in both the lamina propria and detrusor with signs of urothelial ulceration and edema. The differential responses to the treatments were significant (** - p value < 0.01, *** - p value < 0.001). **B)** Bladder muscle expanded in cell culture were subjected to the CPX metabolite, acrolein**,** in the presence or absence of nicotinamide for 0, 2, 4, and 6 hours. Differences in cellular adhesion and cell death is evident in phase contrast images. **C**) FACS quantitation of 7-aminoactinomycin D (*7AAD*) and annexin V staining of bladder detrusor cells were performed in control and in the context of 6 h of acrolein treatment in the presence or absence of nicotinamide.

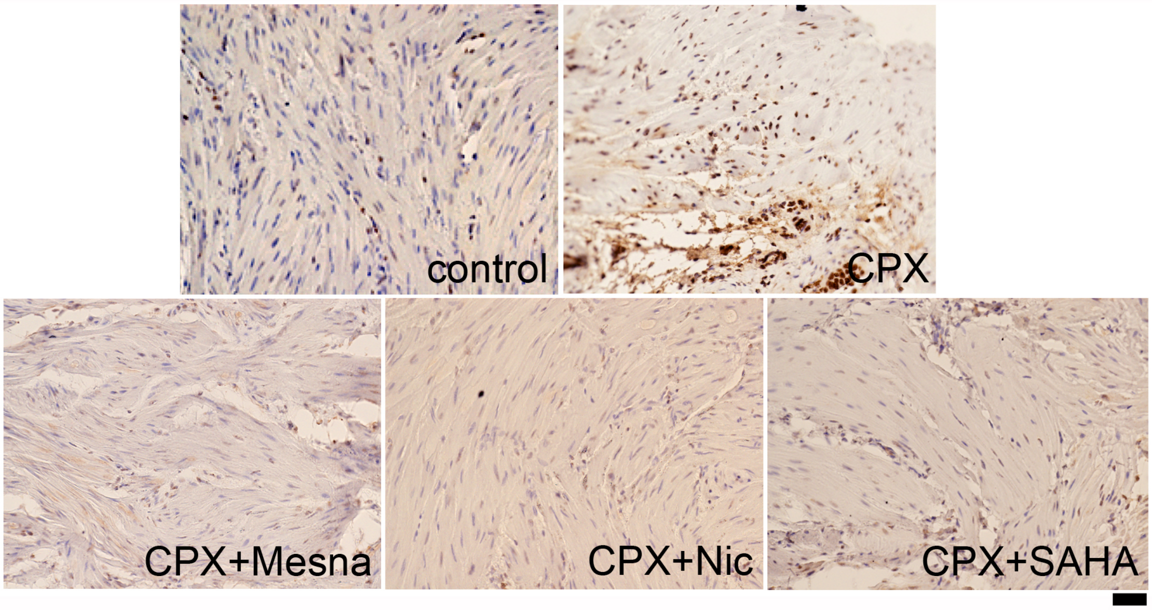

Supplemental Figure 2. **Inflammation associated with bladder muscle imprinting restore by HDAC inhibitor.** Bladders of control and CPX-treated mice in the presence or absence of Mesna (*Mes*) nicotinamide (*nic*) or SAHAwere evaluated for DNA methylation by immune-localization of 5meC expression. The *scale bar* represents 32 µm.
